# Impact of Environmental Uncertainties and Strategic Flexibility in Innovation Activities on NEV Battery Recycling Firms in China

**DOI:** 10.3390/ijerph20043497

**Published:** 2023-02-16

**Authors:** Jingxian Liu, Yingyu Wu, Lili Liu

**Affiliations:** 1 School of Economics and Management, Southeast University, Nanjing 211189, China; 2 Jiangsu Yangtze River Economic Belt Research Institute, Nantong University, Nantong 226002, China; 3 School of International Pharmaceutical Business, China Pharmaceutical University, Nanjing 211189, China

**Keywords:** tech-innovation, uncertainty of environment, firm growth, strategy flexibility, new energy vehicle batteries recycling

## Abstract

Due to the popularization and development of new energy vehicles (NEVs) worldwide, power batteries that have been used are being retired and replaced. In China’s battery recycling industry, the legal NEV battery recycling enterprises are at a negative financial performance. Based on theory of organizational adaptation, the key to innovation performance and sustainable development is recognition of the environment and strengthening organizational flexibility. This study empirically explores the bidirectional dynamic relationships among heterogeneous environmental uncertainties, innovation activities, firm growth and strategic flexibility in Chinese NEV battery recycling firms. A total of 1040 sample data were collected from 2015 to 2021. The research results demonstrate that environmental uncertainty (EU), strategic flexibility (SF) and innovation activities (INNO) all had impacts on firm growth (FG). Specifically, INNO had strongly negative effects in the short term, and in the long term, it will bring a positive effect to FG; the impact of EPU was more important than market uncertainty (MU) to FG and innovation activities. This could be due to the dependence of the Chinese NEV battery recycling industry on government policy. However, MU has a strong impact on SF. Moreover, the levels of SF should be reasonable, otherwise it could be a burden to enterprises. There also exists the bidirectional dynamic relationships between FG and INNO. This study contributes a non-core perspective to strategic flexibility research by revealing the complex environmental mechanism, and to the Chinese NEV battery recycling industry we provide a theoretical basis and practical guidance for government and firms on how to apply SF to promote innovation and realize growth in the present business environment.

## 1. Introduction

As a core component of new energy vehicle development, power batteries will bring serious environmental pollution if they are not disposed of in a standardized way after retirement, so environmentally friendly recycling of retired power batteries has become a pain point that needs to be solved. By December 2021, a total of eight mainland enterprises entered the MIIT’s white-listing. However, according to GGII, which is the largest and most authoritative institution focusing on the research of national strategic emerging industries. Technological innovation is very important to Chinese new energy vehicle (NEV) battery recycling companies.

While technological innovation provides organization brains, management skills provide intelligence and personality. The technological innovation of an enterprise needs to be based on strategic management in order to develop quickly and efficiently [1]. Research related to innovation strategies of firms is of high topicality in the present management literature [2]. Due to increased competition, firms employ various types of innovation activities to position themselves against their competitors. Strategic flexibility has been realized to strengthen this position [3]. Strategic flexibility is a new theory based on the theory of organizational adaptation, which first emerged in the middle 1960s and 1970s. It has evolved from strategy through other disciplines, including management, marketing, innovation, entrepreneurship and operations. In light of these developments, the concept of strategic flexibility has experienced increasing research interest [4,5].

The concept of strategic flexibility is a firm’s capacity to be proactive or respond quickly to changing conditions, with a wide variety of different and intricate environmental uncertainties [6]. However, researchers believe that the strategic flexibility concept is polymorphous in nature, as no fixed standards exist regarding its measurement [7]. Researchers have observed within the literature that “there remains a challenging empirical issue as to how to measure it”. Measurement of innovation-related strategic flexibility refers to resource reconfiguration and coordination [8]. However, there are no studies in the strategic flexibility discipline that capture the capability of strategic flexibility from a combination of financial and non-financial resource reconfiguration. We provide an original contribution to the strategic flexibility literature using this financial–non-financial approach. Specifically, using a capability perspective, we first define strategic flexibility (SF) as consisting of two lower-level capabilities—financial flexibility and non-financial flexibility; we conceptualize financial leverage and asset–liability ratio as “a firm’s capability in financial resource reconfiguration”, and fixed assets ratio and inventory-to-revenue ratio as “a firm’s capability in non-financial resource reconfiguration”.

To successfully innovate, organizations must have the ability to recognize the environment which brings opportunities and threats to firms, and the environmental uncertainty includes a variety of changes in market expectation, customer preference, economic inclination and policy establishment. However, today’s management literature pays attention to innovation performance and economic policy uncertainty [9,10], yet little is known of other uncertainties, such as financial and market uncertainties [11]. Furthermore, China’s domestic energy market shows some unique properties. For example, noble metal in China is showing an increasing price discovery power in recent years, which brings profitable fluctuation in NEV battery recycling market, and Chinese NEV battery recycling is highly policy-oriented. According to the irreversible investment theory, increased energy market uncertainty delays investors’ investment and buyers’ spending on energy products, which then transfers to the NEV battery recycling market fluctuations [12]. Drawing upon the above, it is clear that many concerns in environmental uncertainties related to Chinese NEV battery recycling industry have not been resolved.

In this research, we argue that environmental uncertainty (EU) needs to be classified into market uncertainty and economic policy uncertainty based on its heterogeneity. Thereinto, market uncertainty means the firm’s performance fluctuations [13], any core business activities of enterprises must happen in the market, and the market uncertainty of the commercial market leads to fluctuations in customers and suppliers. It inevitably influences the operating income. The fluctuation of operating income reflects the impact of market uncertainty (MU) on business activities in the market environment. The measurement of EPU is based on Baker ‘s work [14] and it influences economy growth, fiscal revenue and the level of urbanization. Hence, EPU has been the subject of extensive investigation; the scholars’ research topics focus on the impact of EPU on areas such as corporate investment and green innovation [15,16].

To the Chinese NEV battery recycling industry, little attention has been paid to enterprise-level research, especially the legal NEV battery recycling firms. Because these enterprises are the main force in the Chinese NEV battery recycling industry, their performance is significant, and their technical innovation activities present the main innovation level in the Chinese NEV battery recycling industry. In fact, the NEV battery recycling market is not only affected by economic policies but also closely related to the new energy vehicle and noble metal markets. Therefore, a more thorough exploration of uncertainty on the Chinese NEV battery recycling enterprises is necessary.

Moreover, the discrepancy between investment expectations and market performance arises from the scarcity of technology innovation, the chaos of strategic management and shortage of appropriate policies support. Previous literature analyzed the forecast amount of scrap NEV batteries and government subsidy mechanisms [17,18]. Although scholars have shown the importance of strategic flexibility for organizational performance outcomes in different environments, the exact mechanisms and organizational context in the heterogeneous environmental uncertainties are largely unknown. No studies have examined the economic policy uncertainty (EPU) and market uncertainty (MU) simultaneously at the enterprise level. We apply the Panel VAR model to examine the bidirectional dynamic relationships among EPU, MU and innovation performance of NEV battery recycling firms in China. As a dynamic system model, Panel VAR can examine the time-varying interactions among variables.

**Figure 1:**
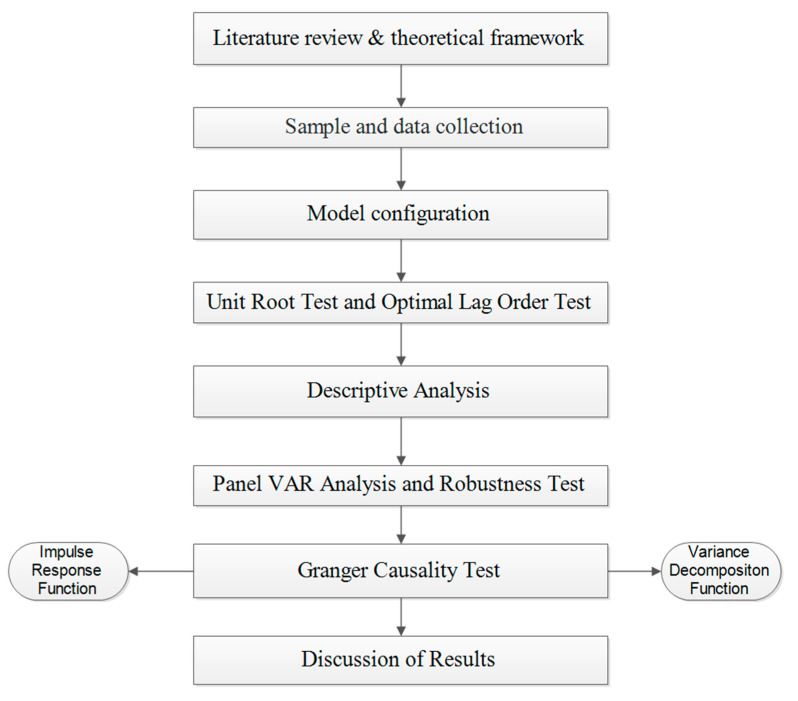
Procedure of the study.

The model does not presuppose causality; all the variables are in the endogenous system and are used as endogenous variables. The specific situation of causality and dynamic interaction is reflected by the actual and objective sample data [19]. The procedure of the study is presented in Figure 1.

Our study contributes to different research streams in environmental uncertainty–strategic flexibility literature. First, we extend strategic flexibility research by providing a new understanding of the performance mechanisms in the context of enterprise innovation behavior.

In more detail, our study explores strategic flexibility as a strategic capability through which enterprise innovation behaviors influence firm performance. Second, we address the research call to examine how the various practices of a firm contribute to strategic flexibility and interplay and result in certain outcomes [20]. The different combinations of aspects related to environmental uncertainty may shape the effectiveness of strategic flexibility. We contribute new insights into the impact of environmental uncertainties as a heterogeneous variable. Third, we develop the measurement of strategic flexibility in innovation activities. Based on real option theory, we treat it as timely resource allocation. Therefore, strategic flexibility is divided into financial resource and non-financial resource. Fourth, we enrich the empirical literature about Chinese NEV battery recycling companies and explain their present situation from the viewpoint of environmental uncertainties based on organizational adaptation theory.

## 2. Literature Review & Theoretical Framework

### 2.1. Environmental Uncertainty

Due to the asymmetric information, financing constraints and unbalanced regional economic development in China, the business environment brings about high uncertainty regarding market and policy and has an important impact on resource investment and innovation activities of firms. Hence, NEV battery recycling firms must take effective actions to deal with the impact of environmental uncertainty [21]. Prior studies have mainly discussed the economic consequences of environmental uncertainty (EU), including market uncertainty (MU) and economic policy uncertainty (EPU). EPU reflects the degree of uncertainty in economic policies, including fiscal policies, monetary policies and regulatory policies [22]. MU reflects the degree of uncertainty in market profit [23]. While the extant literature pays attention to the nexus between EU and firm performance, the studies fail to arrive at a consensus about their relationships.

Some authors argue that EU has a significant economic impact that could be found at the micro-level, where uncertainty is shown to have profound consequences on decision making, and that the “uncertainty effect” leads to incoherent and suboptimal decisions [24]. MU increases the corporate financing pressure and financing costs so that enterprises have higher yield gap [25]. Financial constraints worsen during times of high EPU, whereas funding constraints significantly impede corporate innovation [15]. That is to say, because of high environmental uncertainty, investors require more returns to compensate, so firms will deviate from the optimal investment level by over-investment or under-investment [26].

Contrarily, some authors argue that EU has no impact on macroeconomic performance and even exerts a positive long effect on renewable energy innovation [21], 2022). The negative effect of EPU on trade credit is positively related to firm value [27], and the influences of MU on the equity market could be overall positive and asymmetric across various market circumstances [28]. Furthermore, institutional pressure from environmental uncertainty could bring the application of SF to adopt pro-environmental behaviors [29].

Regarding NEV battery recycling, some studies have investigated the sources of uncertainty in the NEV battery recycling industry [30]; the difference of government policy mechanisms [31] and the government role on consumers’ intentions [32,33]; however, they have not focused on how environmental uncertainty affects the performance and innovation activities of NEV battery recycling firms, and no study has considered the heterogeneities in environmental uncertainty. Therefore, we further discuss the impact of environmental uncertainty on NEV battery recycling firms from the aspects of MU and EPU simultaneously.

### 2.2. Strategic Flexibility Role

With the development of strategic flexibility (SF) theory, its concept becomes more diversified. Nowadays SF is related to other strategic management research topics such as competitive advantage, dynamic ability, response speed and resource allocation efficiency [34,35,36]. In other words, if companies try to maintain a competitive advantage, they have to continuously be one step ahead of their competitors. SF is generated during this continuous procedure. Often SF is also called strategic agility, which reflects a firm’s ability to constantly adapt to the dynamic and unpredictable business environment and refers to “the firm’s ability to integrate, build and reconfigure internal and external competences to address rapidly changing environments” [37].

More specifically, SF is grounded in the concept of competition and uncertainty. Corporate decisions have long-term impacts on how costly it may be for a company to adjust its businesses in the future. How flexibly a business can be adjusted in the future becomes a choice that must be decided in advance [38]. Hence, in an organization, managers should choose one of two repositioning strategies: flexibility or rigidity. The present external environment is realized to be turbulent because of the paradigmatic shifts in globalization, including increasing international conflicts and national wars, COVID-19 compounding global health crises, intensifying digitalization over the last two decades, etc. Before a specific business demand, firms should keep SF to have a free reposition in the future [37]. In some literature, business environment change, either predictable or unpredictable, is generally seen as the trigger of SF by the majority of scholars [1,39]. In summary, the literature claims that firms should develop preparedness to cope with and face environmental uncertainty of complexity, and SF provides a firm with the ability to respond promptly and innovatively to changes in its market environment.

Furthermore, according to the literature, environment changes that can trigger SF may vary, and SF shows its impact as an intervening variable [4,20]. The uncertainties can be the fluctuations and advances in technology. SF mediates the association between entrepreneurial leadership and innovation performance; additionally, SF increases performance in radical innovation both in a certain situation and an environment with technological turbulence [40,41]. To expand firms’ resource pools and establish co-collaboration in innovation activities, SF partially mediates the relationship among the eco-embeddedness position, eco-embeddedness relation, and innovation performance of non-core firms [42]. In the organizations’ mechanism of acquiring external knowledge, SF also plays a mediator role [43].

Investment evaluation of innovative projects is based on net present value (NPV) and other discounted cash flow techniques. These traditional techniques ignore the value of the associated SF. When SF is able to respond to new information from the market and thus increase project value, the use of traditional valuation techniques can go awry. Therefore, appropriate SF can manage well the risk of investing in technological innovation [44]. In dynamic environments, organizational inertia may reduce SF when developing new technologies could change existing resource structures to create new environmental opportunities, and then it is beneficial to generate SF. That is to say, some authors believe that when innovation activities prioritize SF over cost efficiency, innovation capacity may regulate SF [45,46].

### 2.3. Strategic Flexibility and Firm Performance

The dynamic capability theory emphasizes the importance of corporations establishing dynamic capabilities, and the resource-based view is expanded from the static perspective to the dynamic perspective. Company capacity variance affects the difference in firms’ innovation performance [47]; the final goal to keep sustained competitive advantage is the financial performance in the market. Therefore, here come the two questions: whether SF enables a firm’s performance and how SF influences a firm’s performance.

Some authors strongly believe that SF is an important factor affecting a firm’s performance and increasing a firm’s survival time, improving dynamic production and innovation capabilities because it could be triggered in response to competitive actions [48,49]. A higher SF allows companies to rapidly adjust product range, variety, innovativeness and speed of development to capitalize on new opportunities and minimize the negative impact of changing market conditions [50]. Network capability and business model also enhance the positive effect of SF on firm performance [51]. In the Hungarian food industry, the performance affected by SF was evaluated, and it was found that in total, 66.5% of changes in companies’ performance are related to SF [52]. In the textile industry, fashion industry, hotel industry and Chinese sea food industry, SF allow firms to overcome competitive disadvantages and compete effectively against larger firms in the market and has a significant effect on business performance as well [53,54,55,56]. Presently, there is no existing empirical study on strategic flexibility focusing on the new energy vehicle (NEV) batteries recycling industry. Therefore, we further discuss the impact of SF in this industry.

However, some authors hold a negative or skeptical attitude towards the impact of SF on a firm’s performance. The major concern is the unbalanced leverage between SF creation and firm performance. Evidently, proponents highlight that the price to pay for having SF might outweigh its benefits [57]. SF entails higher costs, increased stress among employees and brings a potential lack of focus; managers are distracted from making the long-term commitments needed to implement long-term strategies successfully [58,59]. In other words, a paradoxical challenge emerges for managers seeking to build SF, since this action may generate losses that outweigh potential gains. Therefore, thorough the examination of the SF–performance relationship, SF could have no effect or even negative effects on firm performance [60,61,62].

### 2.4. Conceptual Model

**Figure 2:**
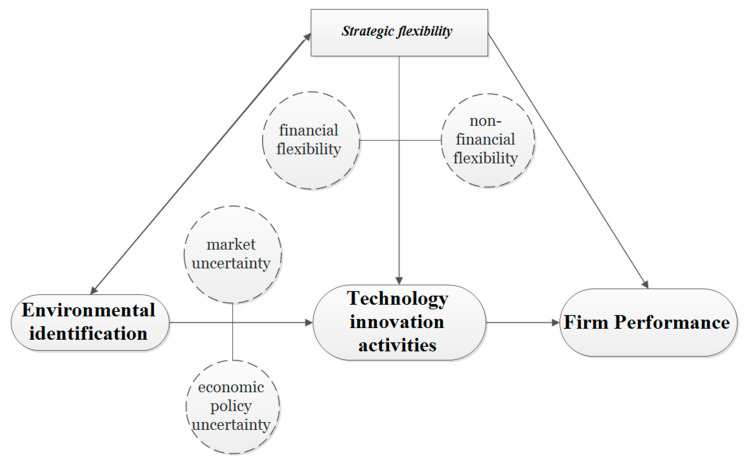
Conceptual model.

From the above analysis, in this research, we focus on two fundamental questions that characterize the dynamic interaction among turbulent environments, firm growth and SF in the Chinese NEV battery recycling industry: Does SF impact firm performance, and how does SF impact firm performance? What factors have an impact on the innovation activities and what kind of environmental uncertainty is more important to firm growth? Figure 2 is the conceptual model of this research.

We argued that a connection could exist between SF and EU, and EU and SF may have impacts on innovation activities. To firm growth (FG), EU could be the barrier, while SF and innovation activities could promote FG. Cultivating SF is an indispensable and favorable factor for enterprises to deal with environmental changes in a dynamic environment. In any stage of the life cycle, companies are restricted by the complicated environment that concerns fierce and stiff competition. From the perspective of real option theory, the reasonable innovation activities could be treated as timely and agile financial and non-financial resource investments. SF builds such support to improve competitiveness and performance in the economic crisis and other environmental uncertainties [63]. SF helps organizations use the market environmental changes to allocate resources (termination or reversion of present resources promise).

Furthermore, SF is not only related to responses to changes in the business environment but also bound with the ability to form and change the environment [58]. The key of SF is how to allocate and assign organization resources in technological innovation activities that play an important role in creating the core competitive value of enterprises. Competitiveness can strengthen SF in turn [64]. Agility and elasticity in SF can help enterprises strengthen their attention to financial management and cost. SF offers solutions for projects adapting to market demand quickly at a low cost and makes companies’ investment efficiency reach optimal results [65]. Regarding the impact of SF on firm growth, building SF could improve companies’ performance in a volatile environment, because SF enhances the identification, integration and allocation of resources, so that the timely application of resources in key innovation activities achieves the firm’s sustainable development [40].

## 3. Research Design

### 3.1. Sample and Data Collection

Combined with the principles of data usability and comparability, the quarterly data of variables: firm growth (FG), market uncertainty (MU), strategic flexibility (SF) and innovation activities (INNO) were obtained from 2015 to 2021 through CSMAR. Variable economic policy uncertainty (EPU) was collected from http://policyuncertainty.com/ (accessed on 22 December 2022). We selected all eight listed Chinese NEV battery recycling companies for the empirical research. The stock codes were, respectively, 002340, 002460, 002594, 002709, 002741, 300409, 600549 and 603799. We set the item “R&D intensity” to measure innovation activities and summarized the FG across two key segments: development ability and profitability, the former was the long-term dimension to measure the development of the firm, and the latter was the short-term dimension to measure the earnings level. FG was measured as the capital accumulation rate, Tobin Q, income growth rate and ROA. SF was divided into two aspects: financial flexibility and non-financial flexibility, and it was measured by financial leverage, asset liability ratio, fixed assets ratio and inventory-to-revenue ratio. MU was measured by sales fluctuation, and EPU was measured by the economic policy index. The basic information of the variables is presented in Table 1.

### 3.2. Empirical Model

The Panel VAR (PVAR) model is a different way in multivariate simultaneous equation systems in macro-econometrics studies to test bidirectional dynamic relationships. This model has various utilization options across economic fields in panel-data settings [69]. PVAR is particularly beneficial to this empirical research because it can deal with the complex relationship between precursor factors and posterior factors. All the variables of the research were in an endogenous system and were used as endogenous variables, so the causal relationship could be bidirectional. The PVAR model was set in the following form: i is enterprise; t means sample selection interval; $y_{\mathrm{it}}$ is the vector composed of endogenous variables for the ith listed company in year t; ${\mathrm{fp}}_{\mathrm{it}}$ is financial performance; ${\mathrm{sf}}_{\mathrm{it}}$ is strategic flexibility; ${\mathrm{mu}}_{\mathrm{it}}$ is market environmental uncertainty; pis the optimal lag length; $a_{i1}$ represents panel fixed effects, $\varepsilon_{\mathrm{it}}$ represents idiosyncratic errors.

$${\mathrm{fg}}_{\mathrm{it}}=a_{i1}+\overset{p}{\underset{j=1}{\sum }}\left(\alpha_{i1}{\mathrm{fg}}_{\mathrm{it}-j}\right)+\overset{p}{\underset{j=1}{\sum }}\left(\alpha_{i1}{\mathrm{sf}}_{\mathrm{it}-j}\right)+\overset{p}{\underset{j=1}{\sum }}\left(\alpha_{i1}{\mathrm{mu}}_{\mathrm{it}-j}\right)+\overset{p}{\underset{j=1}{\sum }}\left(\alpha_{i1}{\mathrm{epu}}_{\mathrm{it}-j}\right)+\overset{p}{\underset{j=1}{\sum }}\left(\alpha_{i1}{\mathrm{inno}}_{\mathrm{it}-j}\right)+\varepsilon_{it}$$

$${\mathrm{sf}}_{\mathrm{it}}=a_{i2}+\overset{p}{\underset{j=1}{\sum }}\left(\alpha_{i2}{\mathrm{fg}}_{\mathrm{it}-j}\right)+\overset{p}{\underset{j=1}{\sum }}\left(\alpha_{i2}{\mathrm{sf}}_{\mathrm{it}-j}\right)+\overset{p}{\underset{j=1}{\sum }}\left(\alpha_{i2}{\mathrm{mu}}_{\mathrm{it}-j}\right)+\overset{p}{\underset{j=1}{\sum }}\left(\alpha_{i2}{\mathrm{epu}}_{\mathrm{it}-j}\right)+\overset{p}{\underset{j=1}{\sum }}\left(\alpha_{i2}{\mathrm{inno}}_{\mathrm{it}-j}\right)+\varepsilon_{it}$$

$${\mathrm{inno}}_{\mathrm{it}}=a_{i3}+\overset{p}{\underset{j=1}{\sum }}\left(\alpha_{i3}{\mathrm{fg}}_{\mathrm{it}-j}\right)+\overset{p}{\underset{j=1}{\sum }}\left(\alpha_{i3}{\mathrm{sf}}_{\mathrm{it}-j}\right)+\overset{p}{\underset{j=1}{\sum }}\left(\alpha_{i3}{\mathrm{mu}}_{\mathrm{it}-j}\right)+\overset{p}{\underset{j=1}{\sum }}\left(\alpha_{i3}{\mathrm{epu}}_{\mathrm{it}-j}\right)+\overset{p}{\underset{j=1}{\sum }}\left(\alpha_{i3}{\mathrm{inno}}_{\mathrm{it}-j}\right)+\varepsilon_{it}$$

### 3.3. Unit Root Test and Optimal Lag Order Test

In estimating the panel data model, it was necessary to test the stationarity of the variables first to avoid spurious regression and ensure accuracy and validity of the results. In this study, on all the series at levels, the popular Levin-Lin-Chu unit-root test, Im-Pesaran-Shin unit-root test and the Fisher-ADF test were performed. The results imply that all series were stationary in levels (Table 2).

After investigating the stability of the variables to be included in the equation, the application of PVAR analysis could be initiated. The first step was to select the optimal lag length. Table 3 identifies the most appropriate delay for analysis. Accordingly, the delay with the smallest value of AIC, BIC and HQIC coefficients was the most appropriate delay. Hence, the length of lag to be used in the study was determined as 5.

## 4. Empirical Analysis and Discussion

Table 4 provides the descriptive statistics of the data used along with their units of measurement. It shows the high standard deviation for the SF variable, and it demonstrates the high volatility of this variable. The minimum and maximum of SF were 0.853 and 179.586, respectively, for the NEV battery recycling firms. The mean of FG was 0.94 with a maximum of 3.710, while the minimum was only 0.212. Additionally, INNO, EPU and MU had 19%, 22.8% and 31.1% volatility. Furthermore, comparing the maximum and minimum of them showed a large distance.

Table 5 shows the empirical results of the PVAR model for the FG, SF and INNO because they were our research objects. SF had a significant positive effect on FG, while EPU had a significant negative effect on it in short term. This result may be attributed to the gap of government policy on the NEV battery recycling industry. SF could also play a moderating role between FG and EPU [7]. As time passed, the impact of EPU showed a positive effect on FG, and this result was consistent with the results that the impact was nonlinear [27].

Another result is the positive and significant effect of innovation activities in the lag 4 period on firm growth, while lag 3 had a different effect on firm growth. This can be due to investment on innovation activities delaying the return. Hence, innovation activities generally involve option decisions in the future [46]. Lagged SF and environmental uncertainty including MU and EPU are important factors in explaining the negative effect on FG in the later period. There could be two reasons to explain the negative effect. Firstly, the expenditure on establishment and application of SF exceeds the expected return value and becomes a burden in firms. Moreover, the negative effect of environmental uncertainty on FG is consistent and broad because environmental uncertainty has a systemic nature so it tends to have a profound impact on all businesses in this industry along the supply chain [27].

Furthermore, to the variable SF, changes in MU had a negative and significant effect on in the first and third lag; and positive effects of MU and INNO existed in the fifth lag. Another result was that EPU and FG did not have a statistically significant effect on SF. This illustrates the necessity of environmental heterogenization when examining the relationship between SF and environmental uncertainty. The results show that, notably, INNO and MU did have strong joint implications to strengthen SF in the latter period.

To the variable INNO, in contrast to the effects on SF, the direct impact of MU on INNO appeared to be weak and statistically insignificant, and EPU was the key to influencing the innovation activities. Moreover, EPU had a positive effect in the first lag and negative effects in the third and fourth lags. That is to say, environmental uncertainty is not always harmful to innovation activities of economies, especially emerging ones [21].

However, most previous empirical studies on firm development and innovation activities have been conducted in developed economies, and there is a dearth of such studies in emerging markets like the Chinese NEV battery recycling industry. Emerging market firms operate in environments characterized by various volatility and uncertainty conditions, such as ambiguous institutions, chaotic supply and demand-side changes [7]. From the real option theory aspect, the external environment has a significant impact on firm growth, because environmental uncertainty may influence these firm’s attitude toward their investments on innovation activities [70]. In the short term, Chinese NEV battery recycling is more likely to hold more cash and increase R&D activities when EPU increases, and the conclusion is the opposite in the long term. From an upper echelon perspective, the impact of environmental uncertainties and strategic flexibility becomes central not only for survival but also for firm growth and success in emerging markets. Though we used Chinese NEV battery recycling firms as a context to investigate our hypotheses, future studies can specifically study the factors that drive and leverage SF.

Overall, the results show that environmental uncertainty (EU), strategic flexibility (SF) innovation activities (INNO) all had impacts on firm growth (FG). Specifically, INNO had strong negative effects in the short term, and in long term it would bring a positive effect to FG. The impact of EPU was more important than market uncertainty (MU) to FG and INNO. This could be due to the dependence of the Chinese NEV battery recycling industry on government policy. However, MU had a strong impact on SF. Moreover, the levels of SF should be reasonable, otherwise it could be useless or a burden to enterprises.

**Figure 3:**
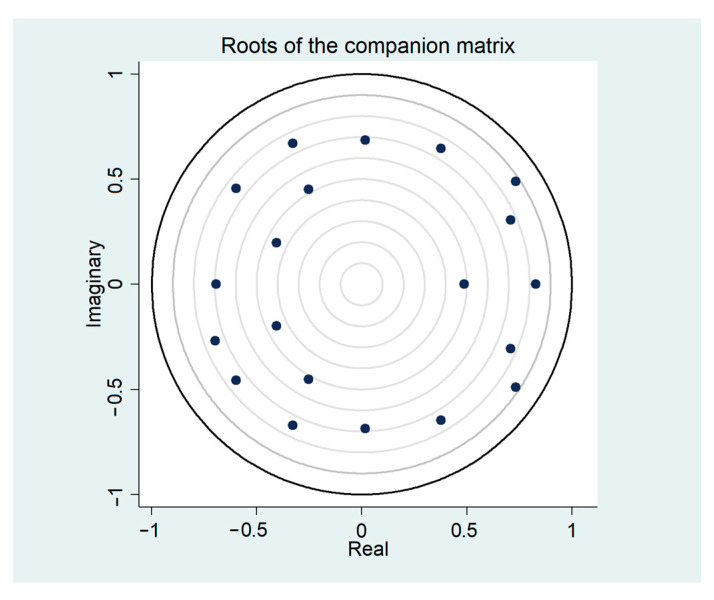
Model stability test result.

Finally, the reliability of the estimates obtained from the vector auto-regression model depended on the stability of the system of equations. Hence, we checked the stability of the system of equations. The stability condition was that all the characteristic roots of the model located within the unit circle (Figure 3). The results indicated that the systems of equations of both groups were stable.

In order to better understand the causal relationship among the variables, the Granger causality test was used in this research. Table 6 expresses the direction of causal relationships when closely examined. While there was a two-way relationship between SF and MU, a unidirectional causal relationship was determined from SF to FG and EPU directions. There was no one-way or two-way causal relationship between EPU and MU variables. A two-way causal relationship was determined from EPU to FG and INNO variables. There were also bidirectional dynamic relationships between FG and INNO.

**Figure 4:**
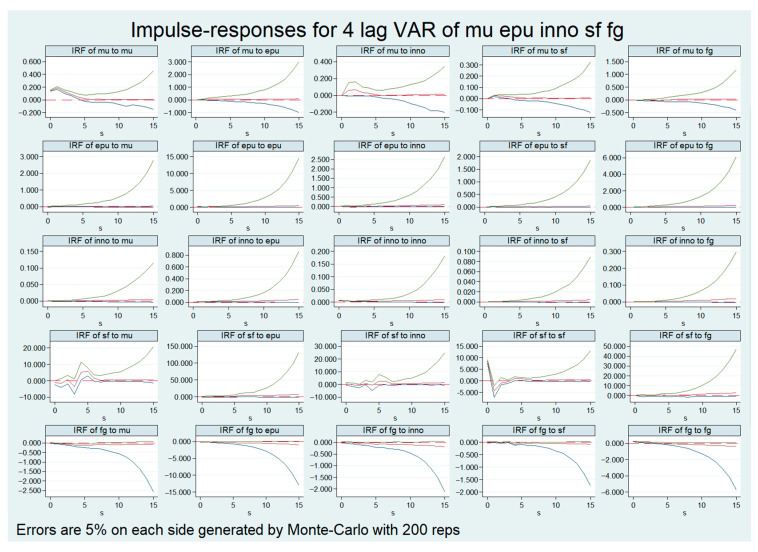
Orthogonalized impulse response function (OIRF).

The impulse response function can measure the current and future effects of other variables generated by the variation of a standard deviation of the random disturbance term, visually display the dynamic interaction between the variables and obtain the empirical basis for determining the time-lag relationship between the variables. Figure 4 shows the results of the impulse response function obtained by simulating 200 times based on the Monte Carlo method and a 95% confidence interval. The first row depicts the accumulated responses of MU to an impulse from the variables. INNO first increased MU, and the effects of these shocks disappeared after an average of five periods. In the fourth row, the accumulated responses of SF seemed more complicated. There was drastic fluctuation from the MU impulse. INNO had a similar effect on SF.

In order to more accurately describe MU, EPU, SF, INNO and FG variables’ interaction effects of the degree, Table 7 displays the results of the variance decomposition analysis for the 1st, 8th and 15th forecast periods. Variance decomposition analysis shows the shocks created by the variables in themselves and other variables by using moving average variances and to what extent they are explanatory of each other. Specifically, SF was self-explanatory by an average of 40.1% in an eight-year period. On the other hand, MU was determined as the variable that explained 39.9% of SF on average. In a 15-year period, interestingly, FG and INNO were greatly affected by EPU. MU contributed about 6.1% and 2.9%, respectively. SF was self-explanatory by an average of 28.6% after MU and EPU came with an average of 30.5%.

## 5. Conclusions

In the current environment of NEV battery recycling in China, achieving innovation is crucial for the survival and development of companies and the creation of competitive advantages. As the business environment becomes increasingly dynamic and complex, it becomes more difficult for Chinese NEV battery recycling firms to achieve sustainable development. Therefore, building strategic flexibility becomes a fundamental way for firms to obtain resources, enhance capabilities and achieve innovation. Based on the organizational adaptation theory, strategic flexibility theory and firm growth theory, this study constructed a theoretical framework of “Environment–strategic flexibility–innovation performance–firm growth” for Chinese NEV battery recycling firms and empirically analyzed the dynamic interaction effectiveness among them. This theoretical model was tested empirically using 1040 samples, and the main findings were as follows.

First, economic policy uncertainty and strategic flexibility apparently had long-term impacts on firm growth, and the impacts of strategic flexibility and economic policy uncertainty were both nonlinear. Innovation activities had a strong impact on firm growth, but the positive effect was delayed. There was no connection between economic policy uncertainty and market uncertainty. Innovation activities were affected more by economic policy uncertainty. Hence, compared with market uncertainty, government attitude and firms’ innovation are more important than other factors to a firms’ sustainable development. Second, the firms adjusted strategic flexibility to market uncertainty, not to economic policy uncertainty; it also explained why the impact of strategic flexibility on firm growth was statistically obvious but not strong. Finally, strategic flexibility also costs, so the level should be controlled appropriately by firms, otherwise it could be useless or unnecessary to enterprises.

## 6. Limitations

This study has limitations that deserve further in-depth exploration in the future. First, due to the data collection limitations, all variables measured in this paper were from Chinese listed NEV battery recycling companies, which may have had subjective bias. Hence, future studies should collect unlisted companies’ data to obtain more accurate research conclusions. Second, this article has not yet distinguished the dimensions of innovation activities. Therefore, future research can choose a different measurement index to explore a differential effect on disparate types of innovation. Third, according to the results, future studies could pay more attention to the relationship between government policy and firm growth in the Chinese NEV battery recycling industry. The balance between cost and return of recycling is really a big issue in recycling firms now. The fluctuant earnings (in particular for graphite, lithium and cobalt) and the recycling channels are the keys of firm performance. Socio-environmental analyses and recycling strategies will be considered in future power batteries’ recycling studies.

## Figures and Tables

**Table 1:** Variable information.

	Level 1	Level 2	Level 3	Source
Variables	Firm Growth	Development ability	Capital accumulation rate	Yufei Zhang et al., 2022 [66]; Rouven E. et al., 2022 [67]
			Tobin Q	
		Profitability	Income growth Rate	
			ROA	
	Strategic Flexibility	Financial flexibility	Financial leverage	Kafetzopoulos, D et al., 2022 [4]; Sanchez, R, 1995 [48]
			Asset liability Ratio	
		Non-financial flexibility	Fixed assets ratio	
			Inventory to revenue ratio	
	Environmental Uncertainty	Market Uncertainty	Sales fluctuation	Stefan Schneck et al., 2022 [27]
		Economic Policy Uncertainty	Economic Policy Index	Jiangfeng Ye et al., 2022 [9]; Deshuai Hou et al., 2022 [10]
	Innovation Activities	Tech-innovation ability	R&D intensity	Andrea and Esteban, 2021 [68]

**Table 2:** Panel VAR unit root results.

Variable	Levin-Lin-Chu Unit-Root Test *p*-Value		Im-Pesaran-Shin Unit-Root Test *p*-Value	
fg	0.0065		0.0025	
sf	0.0000		0.0000	
inno	0.0000		0.0016	
mu	0.0000		0.0009	
	**Fisher-ADF test**			
epu	**Test Statistic**	**1% Ctitical Value**	**5% Critical Value**	**10% Critical Value**
	−6.6450	−4.3800	−3.6000	−3.2400

**Table 3:** Panel VAR lag order selection.

lag	AIC	BIC	HQIC
1	6.4868	7.58963	6.9335
2	1.9731	3.5456	2.6104
3	1.4405	3.5121	2.2807
4	0.5627	3.1660 *	1.6192
5	0.2956 *	3.4669	1.5834 *

* denotes the optimal lag length.

**Table 4:** Descriptive statistics of variables.

Variable	Obs	Mean	Std. Dev.	Min	Max
fg	224	0.94	0.653	0.212	3.710
sf	224	2.509	12.504	0.853	179.586
inno	224	0.016	0.019	0.000	0.060
epu	224	0.414	0.228	0.000	1.000
mu	224	0.104	0.311	0.008	2.864

**Table 5:** Panel VAR results.

Explanatory Lagged Values	Dependent Variable					
	h_ fg		h_ sf		h_ inno	
	Coefficient	*p*	Coefficient	*p*	Coefficient	*p*
L1.h_fg	0.512	0.000 *	−1.657	0.636	0.003	0.313
L1.h_sf	0.003	0.007 *	−1.173	0.000 *	−0.000	0.645
L1.h_inno	−3.740	0.155	−80.457	0.603	0.604	0.000 *
L1.h_epu	−0.442	0.004 *	6.622	0.291	0.031	0.000 *
L1.h_mu	−0.117	0.207	−16.668	0.000 *	−0.003	0.364
L2.h_fg	0.282	0.007 *	−1.305	0.673	−0.004	0.027 **
L2.h_sf	0.002	0.092 ***	−0.871	0.000 *	−0.001	0.015 **
L2.h_inno	−0.999	0.651	55.155	0.681	0.115	0.212
L2.h_epu	0.005	0.968	6.003	0.161	0.005	0.254
L2.h_mu	−0.024	0.682	4.357	0.150	0.002	0.529
L3.h_fg	−0.202	0.017 **	0.064	0.983	−0.001	0.438
L3.h_sf	0.005	0.000 *	−0.642	0.000 *	−0.000	0.370
L3.h_inno	−4.858	0.002 *	27.794	0.753	0.346	0.002 *
L3.h_epu	0.054	0.017 **	5.013	0.176	−0.009	0.007 *
L3.h_mu	−0.079	0.224	−9.832	0.004 *	−0.002	0.540
L4.h_fg	0.097	0.312	−2.223	0.349	0.002	0.268
L4.h_sf	−0.004	0.000 *	−0.374	0.000 *	−0.000	0.334
L4.h_inno	5.632	0.006 *	62.507	0.434	0.086	0.241
L4.h_epu	0.048	0.577	4.450	0.239	−0.008	0.036 **
L4.h_mu	0.004	0.949	−1.573	0.704	0.003	0.101
L5.h_fg	−0.071	0.367	−1.555	0.530	0.002	0.333
L5.h_sf	0.001	0.362	4.274	0.286	0.000	0.394
L5.h_inno	1.030	0.566	155.836	0.029 **	−0.143	0.022 **
L5.h_epu	−0.309	0.002 *	4.274	0.286	−0.004	0.188
L5.h_mu	−0.138	0.028 **	69.673	0.000 *	0.000	0.907

*, **, and *** denote 1%, 5% and 10% statistical significance levels, respectively.

**Table 6:** Granger causality test.

Chi-Square Statistic		Independent Variable				
		fg	inno	sf	epu	mu
Dependent Variable	fg		0.003 *	0.000 *	0.001 *	0.000 *
	inno	0.035 **		0.057 ***	0.000 *	0.032 **
	sf	0.611	0.023 **		0.487	0.000 *
	epu	0.012 **	0.001 *	0.001 *		0.366
	mu	0.243	0.481	0.000 *	0.653	

*, **, and *** denote 1%, 5% and 10% statistical significance levels, respectively.

**Table 7:** Variance decompositions.

Variable	s	MU	EPU	SF	INNO	FG
mu	1	1.000	0.000	0.000	0.000	0.000
epu		0.012	1.000	0.000	0.000	0.000
sf		0.047	0.010	0.943	0.000	0.000
inno		0.001	0.179	0.002	0.818	0.000
fg		0.006	0.074	0.014	0.003	0.903
mu	8	0.714	0.171	0.006	0.094	0.015
epu		0038	0.824	0.014	0.025	0.100
sf		0.399	0.136	0.401	0.061	0.003
inno		0.018	0.763	0.008	0.16 + 2	0.049
fg		0.101	0.652	0.019	0.087	0.141
mu	15	0.640	0.218	0.008	0.096	0.039
epu		0.041	0.769	0.015	0.061	0.115
sf		0.296	0.313	0.286	0.069	0.036
inno		0.029	0.754	0.012	0.112	0.093
fg		0.061	0.717	0.017	0.081	0.124

Variance decomposition: s = 1, 8, 15 percent of variation in the row variable explained by column variable.

## Data Availability

Data are not publicly available due to privacy restrictions. The data that support the findings of this study are available from the author L.L., upon reasonable request.
